# Urinary albumin and 8-oxo-7,8-dihydroguanosine as markers of mortality and cardiovascular disease during 19 years after diagnosis of type 2 diabetes – A comparative study of two markers to identify high risk patients

**DOI:** 10.1016/j.redox.2017.06.005

**Published:** 2017-06-15

**Authors:** Kasper Broedbaek, Rasmus Køster-Rasmussen, Volkert Siersma, Frederik Persson, Henrik E. Poulsen, Niels de Fine Olivarius

**Affiliations:** a Department of Clinical Biochemistry, Rigshospitalet, Copenhagen, Denmark; b Denmark Laboratory of Clinical Pharmacology Q7642, Rigshospitalet, Copenhagen, Denmark; c Department of Clinical Pharmacology, Bispebjerg Hospital, Copenhagen, Denmark; d The Research Unit for General Practice and Section of General Practice, Department of Public Health, University of Copenhagen, Copenhagen, Denmark; e Steno Diabetes Center, Gentofte, Denmark; f Department of Clinical Medicine, Faculty of Health and Medical Sciences, Copenhagen, Denmark

## Abstract

Urinary albumin is an important biomarker used to identify high risk patients with diabetes, but there is a need for new biomarkers that alone or in combination with urinary albumin could give an even better prediction of clinical patient outcomes. One promising biomarker is 8-oxo-7,8-dihydroguanosine (8-oxoGuo) that represents intracellular oxidative stress. We investigated the ability of microalbuminuria (MA) and urinary 8-oxoGuo, alone and in combination, to predict mortality and cardiovascular disease (CVD) in patients with type 2 diabetes.

We used data from 1381 newly diagnosed diabetes patients, and urinary albumin and 8-oxoGuo were assessed in morning urine collected at the time of diabetes diagnosis and at a follow-up visit 6 years later. Associations between the urinary markers and mortality and CVD were assessed in Cox proportional hazards regression models. Test performance was assessed using sensitivity, specificity, positive predictive value and negative predictive value for 10-year mortality and 10-year incidence of CVD.

Both 8-oxoGuo and urinary albumin were statistically significantly associated with all-cause mortality at diagnosis as well as at 6-year follow-up. At diagnosis only urinary albumin was associated with CVD. In contrast, only 8-oxoGuo was associated with CVD at 6-year follow-up. When investigating test performance, we found that by combining information from MA and 8-oxoGuo the ability to correctly identify patients at risk could be improved.

The findings suggest that measurement of urinary 8-oxoGuo provides additional information about risk to that obtained from urinary albumin, and that the combined use of 8-oxoGuo and urinary albumin could be useful for a better identification of patients at risk of CVD and death.

## 1 Introduction

Microalbuminuria (MA) is associated with an increased risk of cardiovascular disease (CVD) [1], renal disease and death [2], and in patients with type 2 diabetes (T2DM) MA correlates positively with the degree of coronary atherosclerosis [3]. MA is caused by an increased transglomerular passage of albumin and this passage is most likely mediated by an increase in filtration pressure and damage to the glomerular barrier. People with MA have an increased transcapillary escape rate, which indicates that MA is not just a marker of renal injury but also of systemic injury and dysfunction of the endothelium of the blood vessels [4]. Furthermore, an increased transvascular lipoprotein transport has been demonstrated in patients with T2DM and MA, which could contribute to the formation of atherosclerosis [5]. The relationship between MA and changes in the vessel wall is not yet fully understood, but there are several hypotheses, including destruction of the glycocalyx on the surface of the glomerular endothelium [6], association with inflammatory processes, endothelial dysfunction and platelet activation [7].

Urinary albumin is an important biomarker used in the identification of high risk diabetes patients, but there is a need for new biomarkers that alone or in combination with urinary albumin could give an even better prediction of clinical patient outcomes. New biomarkers that can detect an accelerated rate of complications to T2DM earlier than MA are warranted to ensure initiation of more aggressive treatment regimens in high risk patients early in the natural history of the disease [8], [9]. One promising biomarker is the oxidative stress marker 8-oxo-7,8-dihydroguanosine (8-oxoGuo) that represents intracellular oxidative stress [10], [11]. Oxidative stress is traditionally related to a situation where the formation of reactive oxygen species exceeds the capacity of an organism's antioxidant defense system. Furthermore, it is now clear that changes in oxidative status have profound effects on a number of signaling and regulatory processes [12]. Oxidative stress is thought to play a pivotal role in the development of diabetic complications, and several possible mechanisms have been suggested, including increased formation of advanced glycation end products (AGEs), activation of protein kinase C and hexosamine pathways, and damage to nucleic acids [13].

The RNA oxidation marker 8-oxoGuo is considered to be a product of intracellular oxidative reactions. High urine concentrations of 8-oxoGuo at diagnosis, 6 years later and an increase in this marker over that period were associated with increased mortality in patients with T2DM [10], [11]. Whether the ability of 8-oxoGuo to identify patients who are at high risk of complications is equal to or even greater than that of urinary albumin is not known, and it is also not known if the combined use of both biomarkers provides better prognostic information. Because urinary albumin and 8-oxoGuo reflect different pathological processes, the combined use could provide more information compared with that obtained from a single biomarker.

In this study, we investigated the ability of MA and urinary 8-oxoGuo, alone and in combination, to predict CVD and mortality in patients with T2DM.

## 2 Methods

### 2.1 Study population

In the Diabetes Care in General Practice (DCGP) study [14], 474 general practitioners agreed to prospectively include all subjects on their practice list who fulfilled the following criteria: newly diagnosed diabetes based on hyperglycaemic symptoms and/or raised blood glucose values, diagnosed between 1 March 1989 and 28 February 1992, and aged 40 years or over. The diabetes diagnosis was subsequently confirmed with a single fasting whole blood/plasma glucose value of ≥7.0/8.0 mmol/l, measured at a major laboratory. The protocol-based exclusion criteria were life-threatening somatic disease, severe mental illness, or unwillingness to participate. Accordingly, 162 patients were excluded and the study population consisted of 1381 newly diagnosed diabetes patients. Based on the onset of insulin treatment within 180 days of diagnosis, approximately 97.5% were considered to have T2DM [14]. One freshly voided morning urine sample was collected from all patients at the time of diagnosis and on average 6 years later.

The protocol was approved by the ethics committee of Copenhagen and Frederiksberg and informed consent was obtained from all patients.

### 2.2 Assessment of urinary markers and covariates

Urinary albumin and 8-oxoGuo were measured in a freshly voided morning urine sample. Measurement of urinary albumin was done using a polyethyleneglycol radioimmunoassay [15]. The frozen urine samples were assayed between 2009 and 2010 for 8-oxoGuo using a validated method of ultraperformance liquid chromatography (UPLC) and tandem mass spectrometry [16]. Both urinary albumin and 8-oxoGuo were normalized against urinary creatinine concentration.

The assessment of the remaining patient characteristics at baseline and at 6-year follow-up has been described elsewhere [14]. Cohabitation status, education level and height (used in BMI calculation) were only assessed at diagnosis.

### 2.3 Ascertainment of outcomes

Information on all-cause mortality and cardiovascular morbidity was obtained from the Danish Civil Registration System, the Danish National Patient Register, and the Danish Register of Causes of Death [17], [18], [19]. The patients were followed up in these registers until 1 January 2009, for a total of 19 years after the diagnosis (or 13 years after the 6-year assessment of the urinary markers). Cardiovascular morbidity was defined as incidence of a fatal or non-fatal myocardial infarction stroke, or peripheral vascular disease. The vital status of one patient could not be assessed because this person had emigrated in 1992.

### 2.4 Statistical analysis

The characteristics of the patients at diagnosis and – for those that were still in the study at that time point – at 6-year follow-up are shown as median with inter-quartile range (IQR) for continuous characteristics, or number with percentage for categorical characteristics. The patients were categorized into four groups according to renal involvement (MA, U-albumin/U-creatinine ≥ 2.5 mg/mmol) and oxidative stress (U-8-oxoGuo/U-creatinine ≥ the median at diagnosis and 6-year follow-up, respectively). Kaplan-Meier curves illustrate the mortality and CVD incidence following diabetes diagnosis and 6-year follow-up examination, respectively, for these four groups.

Associations between 8-oxoGuo and urinary albumin, and mortality and CVD incidence were assessed in Cox proportional hazards regression models. These associations were estimated for the four-class variable as described above, and by the continuously valued U-8-oxoGuo/U-creatinine and U-albumin/U-creatinine variables, both separately and jointly. For each association assessment two models were estimated: a model only adjusted for age, sex and diabetes duration (for the assessment at 6-year follow-up), and a second model further adjusted for education, cohabitation status, smoking status, triglycerides, total cholesterol, hypertension, hemoglobin A_1c_, serum creatinine, BMI, physical activity, presence of retinopathy (yes/no) and CVD (yes/no) at baseline (for analyses on mortality).

Sensitivity, specificity, positive predictive value (PPV) and negative predictive value (NPV) were calculated for the above defined dichotomisations of urinary albumin and 8-oxoGuo for 10-year mortality and 10-year occurrence of CVD. Reported *p* values were two-sided and *p* < 0.05 was considered to be significant. Analyses were performed with SAS version 9.4.

## 3 Results

### 3.1 Patient characteristics

Table 1 shows characteristics of the study participants. Data from 1.381 patients with newly diagnosed T2DM were included at baseline and 970 participated in the 6-year follow-up. 312 patients died in the period from baseline to 6-year follow-up. The median age (IQR) at diagnosis of was 65.4 (55.7–73.6) years, with a slight male preponderance (53%). The median age at 6-year follow-up was 69.2 (59.9–77.3) years, with an equal distribution of men and women (51.4% and 48.6%, respectively). 31.7% had MA at diagnosis, and 34.7% had MA at 6-year follow-up. 4.3% and 5.4% had macroalbuminuria (> 25 mg/mmol) at diagnosis and at 6-year follow-up, respectively. The proportion of patients with retinopathy, peripheral neuropathy and CVD increased from diagnosis to 6-year follow-up (4.5–14.9%, 19.3–25.6%, 12.7–17%, respectively). At 6-year follow-up 57.5% were treated with oral antidiabetic drugs, 29.9% with diet alone and 12.6% with insulin. Median HbA_1c_ was 10.2% at diagnosis and 8.6% (reference interval: 5.4–7.4% at the time of the study) at 6-year follow-up.

**Table 1:** Characteristics of patients at diagnosis of type 2-diabetes and at 6-year follow-up.

	At diabetes diagnosis	At 6-year follow-up
N	1381	970
U-albumin/U-creatinine (mg/mmol), median (IQR)	1.30 (0.68–3.25)	1.55 (0.75–3.78)
Microalbuminuria (U-albumin/U-creatinine ≥ 2.5 mg/mmol), n (%)	418 (31.7)	315 (34.7)
U-8-oxoGuo/U-creatinine (nmol/mmol), median (IQR)	3.64 (2.86–4.77)	3.65 (2.94–4.66)
Age (years), median (IQR)	65.4 (55.7–73.6)	69.2 (59.9–77.3)
Male sex, n (%)	733 (53.1)	492 (50.7)
Diabetes duration (years), median (IQR)	–	5.71 (5.01–6.32)
Basic education only, n (%)	1033 (78.8)	729 (78.1)
Living alone, n (%)	434 (32.2)	320 (35.4)
Smoking status, n (%)		
Never	405 (30.1)	296 (33.0)
Previous	469 (34.8)	323 (36.0)
Current	472 (35.1)	278 (31.0)
Fasting triglycerides (mmol/L), median (IQR)	1.98 (1.41–2.91)	1.80 (1.24–2.62)
Total cholesterol (mmol/L), median (IQR)	6.2 (5.4–7.1)	6.0 (5.3–6.8)
Hypertension, n (%)	1026 (74.3)	709 (73.1)
Hemoglobin A1c (%), median (IQR)	10.2 (8.7–11.8)	8.6 (7.8–9.8)
Serum creatinine (µmol/L), median (IQR)	89 (80–101)	90 (80–103)
BMI (kg/m^2^), median (IQR)	29.1 (26.1–32.7)	28.4 (25.5–32.0)
Physical activity, n (%)		
Inactive	372 (27.7)	265 (29.6)
Active	973 (72.3)	630 (70.4)
Anti-diabetes treatment, n (%)		
Diet only		290 (29.9)
Oral anti-diabetes treatment		557 (57.5)
Insulin		122 (12.6)
Retinopathy, n (%)	55 (4.5)	127 (14.9)
Peripheral neuropathy, n (%)	263 (19.3)	241 (25.6)
Cardiovascular disease, n (%)	176 (12.7)	164 (17.0)

### 3.2 Urinary 8-oxoGuo and albumin and risk of CVD and death

We identified a total of 966 deaths and 613 incident cardiovascular events during the 19 years of follow-up.

**Fig. 1:**
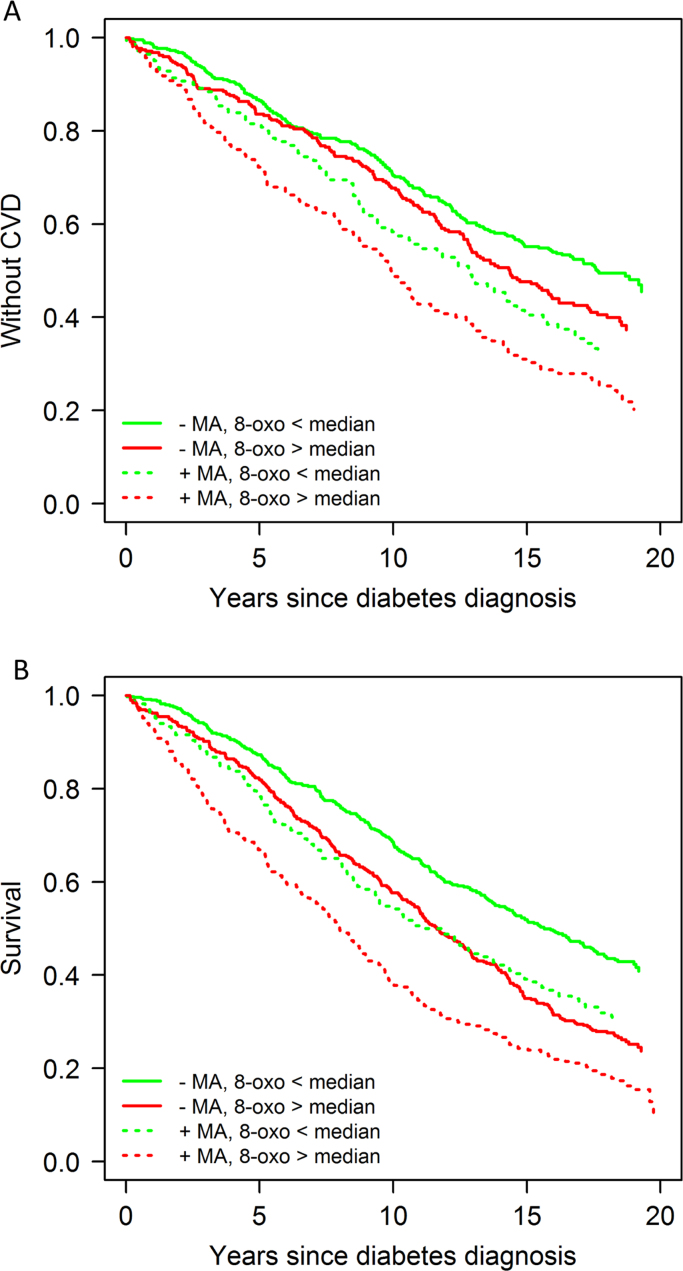
Kaplan–Meier estimates of incidence of CVD and death for all subjects according to the levels of urinary 8-oxoGuo (below or above median) and albumin (presence or absence of MA) at diabetes diagnosis. Both urinary albumin and 8-oxoGuo were normalized against urinary creatinine concentration.

**Fig. 2:**
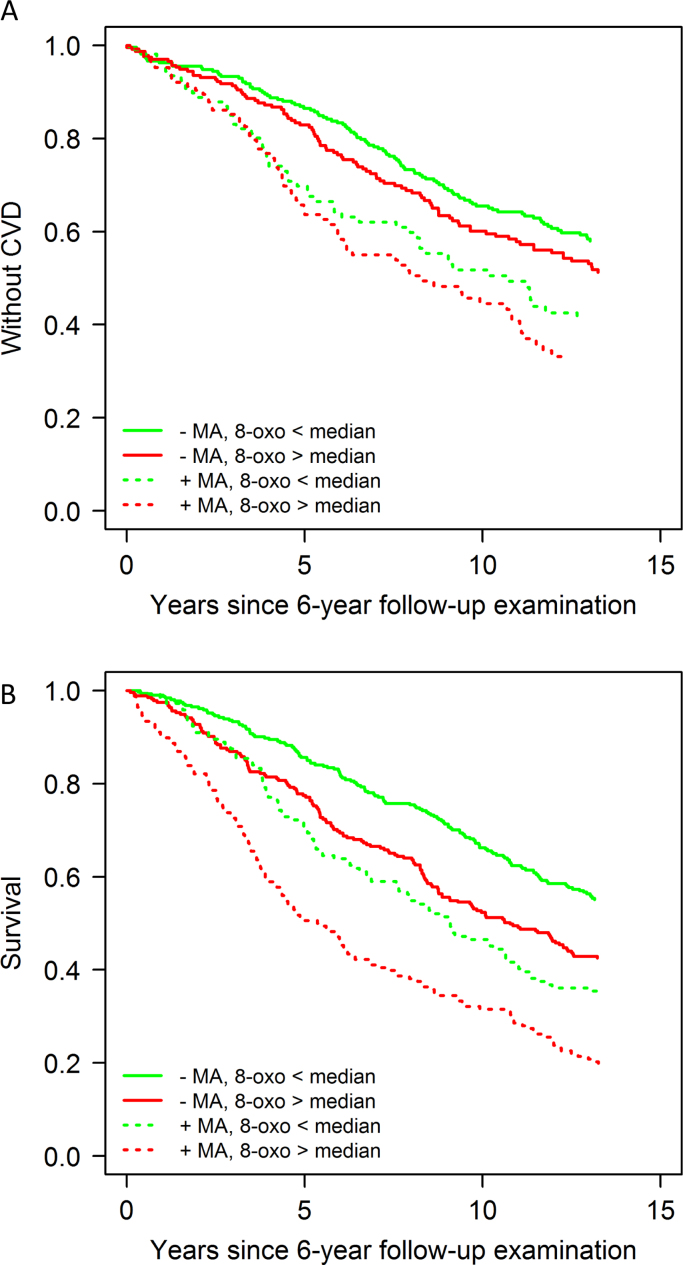
Kaplan–Meier estimates of incidence of CVD and death for all subjects according to the levels of urinary 8-oxoGuo (below or above median) and albumin (presence or absence of MA) at 6-year follow-up. Both urinary albumin and 8-oxoGuo were normalized against urinary creatinine concentration.

Kaplan-Meier estimates of CVD and death for all subjects according to the levels of urinary 8-oxoGuo (below or above median) and urinary albumin (presence or absence of MA) are shown in Fig. 1 and Fig. 2. The presence of MA and 8-oxoGuo above the median identified a group of patients with a higher mortality and CVD morbidity compared with patients without these risk factors, and patients that were risk marker discordant (MA but 8-oxoGuo below median or no MA but 8-oxoGuo above median) had intermediate risk.

These results were corroborated by the Cox regression analyses where the combined categorical information of presence/absence of MA and 8-oxoGuo above/below the median was robustly associated with the outcomes independent of the covariates (Tables 2a and 2b).

**Table 2a:** Association of the combined categorical information of presence/absence of MA and 8-oxoGuo above/below the median at the time of diagnosis with 19-year mortality and CVD morbidity among patients with T2DM.

	Model 1 HR (95% CI)	P value	Model 2 HR (95% CI)	P value
All-cause mortality				
-MA, 8-oxoGuo < median	1.00		1.00	
-MA, 8-oxoGuo > median	1.10 (0.92–1.31)	0.30	1.08 (0.88–1.32)	0.49
+MA, 8-oxoGuo < median	1.42 (1.15–1.74)	0.001	1.18 (0.92–1.52)	0.20
+MA, 8.oxoGuo > median	1.69 (1.39–2.05)	<0.0001	1.59 (1.27–1.99)	<0.0001
CVD				
-MA, 8-oxoGuo < median	1.00		1.00	
-MA, 8-oxoGuo > median	1.00 (0.80–1.25)	1.00	0.94 (0.72–1.23)	0.66
+MA, 8-oxoGuo < median	1.57 (1.22–2.03)	0.0005	1.33 (0.96–1.83)	0.082
+MA, 8.oxoGuo > median	1.63 (1.28–2.09)	<0.0001	1.50 (1.12–2.03)	0.007

Model 1: adjusted for age and sex. Model 2: adjusted for age, sex, education, cohabitation status, smoking status, triglycerides, total cholesterol, hypertension, hemoglobin A_1c_, serum creatinine, BMI, physical activity, presence of retinopathy (yes/no) and CVD at baseline (for analyses on mortality).

**Table 2b:** Association of the combined categorical information of presence/absence of MA and 8-oxoGuo above/below the median at 6- year follow-up with 13 years mortality and CVD morbidity among patients with T2DM.

	Model 1 HR (95% CI)	P value	Model 2 HR (95% CI)	P value
All-cause mortality				
-MA, 8-oxoGuo < median	1.00		1.00	
-MA, 8-oxoGuo > median	1.34 (1.09–1.67)	0.007	1.48 (1.14–1.92)	0.003
+MA, 8-oxoGuo < median	1.74 (1.37–2.22)	<0.0001	1.33 (0.97–1.83)	0.08
+MA, 8.oxoGuo > median	2.20 (1.70–2.84)	<0.0001	2.41 (1.77–3.28)	<0.0001
CVD				
-MA, 8-oxoGuo < median	1.00		1.00	
-MA, 8-oxoGuo > median	1.15 (0.90–1.47)	0.28	1.10 (0.81–1.49)	0.54
+MA, 8-oxoGuo < median	1.62 (1.18–2.23)	0.003	1.21 (0.81–1.81)	0.35
+MA, 8.oxoGuo > median	1.75 (1.29–2.37)	0.0003	1.71 (1.20–2.45)	0.003

Model 1: adjusted for age, sex and diabetes duration. Model 2: adjusted for age, sex, education, cohabitation status, smoking status, triglycerides, total cholesterol, hypertension, hemoglobin A_1c_, serum creatinine, BMI, physical activity, presence of retinopathy (yes/no) and CVD at baseline (for analyses on mortality).

The associations between urinary 8-oxoGuo and urinary albumin and all-cause mortality and CVD were analyzed individually in regression analyses, where 8-oxoGuo and albumin were included as continuous variables (Tables 3a and 3b).

**Table 3a:** Relationship of urinary albumin and 8-oxoGuo with CVD and death.

(a) 19 years HR for the outcomes based on risk factor values at diagnosis.							
		Model 1			Model 2		
		HR (95% CI)	p-value	p-value^a^	HR (95% CI)	p-value	p-value^a^
**Death**							
Individually							
	Albumin	1.03 (1.02–1.04)	< 0.0001		1.03 (1.01–1.05)	0.005	
	8-oxoGuo	1.26 (1.07–1.48)	0.005		1.68 (1.15–2.46)	0.007	
Jointly^b^							
	Albumin	1.03 (1.02–1.04)	< 0.0001	< 0.0001	1.03 (1.01–1.05)	0.004	0.08
	8-oxoGuo	1.15 (0.97–1.37)	0.10		1.65 (1.09–2.51)	0.018	
**CVD**							
Individually							
	Albumin	1.03 (1.02–1.05)	< 0.0001		1.04 (1.03–1.06)	< 0.0001	
	8-oxoGuo	1.39 (0.92–2.11)	0.12		1.27 (0.75–2.15)	0.38	
Jointly^b^							
	Albumin	1.03 (1.02–1.05)	< 0.0001	0.98	1.04 (1.03–1.06)	< 0.0001	0.30
	8-oxoGuo	1.35 (0.88–2.06)	0.16		1.24 (0.72–2.14)	0.43	

Model 1: adjusted for age, sex and diabetes duration. Model 2: adjusted for age, sex, education, cohabitation status, smoking status, triglycerides, total cholesterol, hypertension, hemoglobin A1c, serum creatinine, BMI, physical activity, presence of retinopathy (yes/no) and CVD at baseline (for analyses on mortality).

a For the interaction between 8-oxoGuo and urinary albumin.

b Both albumin and 8-oxoGuo are included in the model.

**Table 3b:** Relationship of urinary albumin and 8-oxoGuo with CVD and death.

(b) 13 years HR for the outcomes based on risk factor values measured 6 years after diagnosis.							
		Model 1			Model 2		
		HR (95% CI)	p-value	p-value^a^	HR (95% CI)	p-value	p-value^a^
**Death**							
Individually							
	Albumin	1.04 (1.02–1.07)	0.001		1.04 (1.00–1.08)	0.03	
	8-oxoGuo	1.71 (1.30–2.26)	0.0001		1.79 (1.34–2.38)	< 0.0001	
Jointly^b^							
	Albumin	1.04 (1.01–1.06)	0.003	1.00	1.03 (1.00–1.07)	0.08	0.06
	8-oxoGuo	1.60 (1.24–2.07)	0.0003		1.66 (1.26–2.18)	0.0003	
**CVD**							
Individually							
	Albumin	1.03 (1.00–1.06)	0.09		1.03 (0.98–1.09)	0.28	
	8-oxoGuo	1.52 (1.21–1.92)	0.0004		1.53 (1.21–1.94)	0.0004	
Jointly^b^							
	Albumin	1.03 (1.00–1.06)	0.10	0.007	1.03 (0.97–1.08)	0.35	0.41
	8-oxoGuo	1.41 (1.14–1.75)	0.002		1.38 (1.08–1.77)	0.01	

Model 1: adjusted for age, sex and diabetes duration. Model 2: adjusted for age, sex, education, cohabitation status, smoking status, triglycerides, total cholesterol, hypertension, hemoglobin A1c, serum creatinine, BMI, physical activity, presence of retinopathy (yes/no) and CVD at baseline (for analyses on mortality).

a For the interaction between 8-oxoGuo and urinary albumin.

b Both albumin and 8-oxoGuo are included in the model.

When assessed at diagnosis both 8-oxoGuo and urinary albumin were associated with mortality (Table 3a). The multivariably adjusted hazard ratios for all-cause mortality per unit increase in 8-oxoGuo and urinary albumin were 1.68 (95% CI, 1.15–2.46; *P* = 0.007) and 1.03 (95% CI, 1.01–1.05; *P* = 0.005) respectively. The estimates were largely unchanged when both urinary albumin and 8-oxoGuo were included in the models. Analyses using values of 8-oxoGuo and albumin from 6-year after diagnosis yielded similar results (Table 3b). There was an interaction between 8-oxoGuo and urinary albumin when assessed at diagnosis in the age and sex adjusted model, but not in the multivariably adjusted model. There were no significant interactions between 8-oxoGuo and urinary albumin assessed at 6-year follow-up.

At diagnosis urinary albumin was significantly associated with CVD (multivariably adjusted hazard ratio 1.04; 95% CI 1.03–1.06; *P* < 0.0001), while 8-oxoGuo from the same sample was not (Table 3a). In contrast, 8-oxoGuo measured at 6-year follow-up was significantly associated with CVD (multivariably adjusted hazard ratio 1.53; 95% CI 1.21–1.94; *P* = 0.0004), while urinary albumin was not (Table 3b). The estimates did not change much when both urinary albumin and 8-oxoGuo were included in the models. Regarding the risk for CVD, there were no interactions between 8-oxoGuo and urinary albumin when assessed at diagnosis. For values assessed at 6 years there was an interaction between the terms in the age and sex adjusted model, but not in the multivariably adjusted model.

### 3.3 Test performance for 10-year all-cause mortality and CVD

In Table 4 the ability of MA and 8-oxoGuo ≥ median to predict 10-year all-cause mortality and CVD is shown with the use of sensitivity, specificity, PPV and NPV. For 10-year all-cause mortality and CVD the specificity of MA was high (71.7–75.4%), both when assessed at diagnosis and at 6-year follow-up, meaning that absence of MA has a fair ability to correctly identify those patients what will not develop CVD or die within the next 10 years. In contrast, the ability of MA to correctly identify those that will develop CVD or die within the next 10 years was poor (sensitivity 38.2–45.0%).

**Table 4:** Test performance of MA and 8-oxoGuo>median in patients with T2DM.

At diagnosis:												
	MA				High 8-oxoGuo				MA AND high 8-oxoGuo			
	Sens	Spec	PPV	NPV	Sens	Spec	PPV	NPV	Sens	Spec	PPV	NPV
10 year	41.6%	75.4%	55.3%	63.9%	58.9%	56.9%	50.7%	64.8%	28.0%	87.3%	61.8%	62.4%
All-cause mortality												
10 year CVD	39.7%	73.4%	41.8%	71.7%	53.1%	52.3%	35.2%	69.6%	25.3%	84.8%	44.6%	70.2%

At 6-year follow-up:												
	MA				High 8-oxoGuo				MA AND high 8-oxoGuo			
	Sens	Spec	PPV	NPV	Sens	Spec	PPV	NPV	Sens	Spec	PPV	NPV
10 year	45.0%	74.7%	61.9%	59.8%	58.3%	57.9%	56.8%	59.4%	26.8%	88.8%	68.5%	57.2%
All-cause mortality												
10 year CVD	38.2%	71.7%	43.9%	66.7%	52.1%	51.5%	39.4%	63.9%	20.8%	84.7%	44.2%	64.8%

Sensitivity, specificity, positive predictive value (PPV) and negative predictive value (NPV) of MA and 8-oxoGuo > median for 10-year mortality and 10-year occurrence of CVD.

When using the median of 8-oxoGuo as a cut-off, it was shown that the sensitivity and specificity regarding 10-year all-cause mortality and incidence of CVD were of the same magnitude, both when assessed at diagnosis and at 6-year follow-up (sensitivity 52.1–58.9%, specificity 51.5–57.9%).

Combining MA and 8-oxoGuo ≥ median increased specificity compared with MA alone (specificity 84.7–88.8%). Thus, absence of MA plus 8-oxoGuo < median had a good ability to correctly identify those patients that would not develop CVD or die within the following 10 years. In contrast, the ability of MA combined with 8-oxoGuo ≥ median to correctly identify those that will develop CVD or die within the next 10 years was poor (sensitivity 20.8–28.0%). Compared to only using MA to stratify the patients an increase in specificity was obtained by combining MA and 8-oxoGuo, which was most pronounced for 10-year all-cause mortality (specificity 75.4–87.3%) when markers were assessed at diagnosis.

The PPVs in Table 4 are the probabilities of developing CVD or die within the next 10 years when the test result was positive (MA was present or 8-oxoGuo > median), and the NPVs are the probabilities of not developing CVD or die within the next 10 years when the test was negative.

These predictive values should be considered together with the general 10-year incidence of CVD and death in the population (from diagnosis: death 43.3%, CVD 32.8%; from 6-year follow-up: death 49.4%, CVD 37.8%). Thus, in this setting (in this population and with an arbitrary 8-oxo-Guo ≥ median cut off) both tests alone were clearly better than chance to positively predict mortality (from diagnosis: 8-oxoGuo PPV 50.7% and MA PPV 55.3% versus chance 43.3%; from 6-year follow-up: 8-oxoGuo PPV 56.8% and MA PPV 61.9% versus chance 49.4%), and the combined test was rather convincingly able to predict death (from diagnosis: PPV 61.8% versus chance 43.3%; from 6-years follow-up: PPV 68.5% versus chance 49.4%). MA was slightly better than 8-oxoGuo to predict the occurrence of CVD. The predictive power of being alive 10 years after a negative test result was equally good for MA and 8-oxoGuo, but the negative predictive power regarding CVD was not substantially better than chance for any of the tests. Combining the tests did not improve NPV.

## 4 Discussion

This is the first study to investigate the combined use of the two non-invasive biomarkers urinary albumin and urinary 8-oxoGuo for identifying high risk patients with T2DM. We found that both MA and urinary 8-oxoGuo individually were predictors of mortality in T2DM patients, and when using them in combination the ability to identify high-risk patients increased, indicating an added clinical value of 8-oxoGuo as supplement to the present use of MA.

Our analyses showed that when analyzed individually in a Cox proportional hazards regression analysis, both 8-oxoGuo and urinary albumin were associated with all-cause mortality at diagnosis as well as at 6-year follow-up. At diagnosis only urinary albumin was associated with CVD. In contrast, only 8-oxoGuo was associated with CVD at 6-year follow-up. Although this difference between the results at diagnosis and at follow-up could be a chance finding, it could also reflect that some factors are important at the time of diagnosis, i.e. in the untreated state, as opposed to the situation when the diabetes has been present for 6 or more years with various treatments, among which blockers of the renin-angiotensin system are frequently used and known to reduce levels of urinary albumin [20]. Significant interactions between 8-oxoGuo and urinary albumin were shown in the age and sex adjusted models, but not in the multivariably adjusted models for all-cause mortality at diagnosis and for CVD at 6-year follow-up. Thus, for these analyses greater emphasis should be placed on the results derived from the multivariably adjusted models.

Our results are in accordance with previous studies of diabetes patients that have found associations between albuminuria and incidence of CVD and mortality [21], [22], [23], [24], [25], [26], [27], [28], [29]. The associations between urinary 8-oxoGuo and CVD and mortality have so far only been investigated in the present cohort. By using urinary 8-oxoGuo and urinary albumin together the patients can be classified in risk categories according to the combination of presence or absence of MA and high or low levels of 8-oxoGuo. When investigating the sensitivity, specificity, PPV and NPV in relation to 10-year mortality and 10-year CVD, we found that by combining information on MA and 8-oxoGuo the ability to correctly identify patients at risk could be increased.

Although the association between MA and mortality in type 2 diabetes is well-established [27], [30], [31], few studies have investigated test performance of MA using sensitivity, specificity, PPV and NPV in the prediction of CVD and death. In a study by Rutter et al., 86 patients with T2DM (43 patients with MA (urinary albumin excretion rate > 10.5–200 g/min) individually matched with 43 normoalbuminuric patients) the relationship between MA and future coronary heart disease was investigated [32]. Mean (SD) follow-up length was 2.5 (0.9) years. The authors found the following test performance in the prediction of coronary events: sensitivity: 78%, specificity: 53%, PPV: 16%, and NPV: 95%. This is in contrast to our study that for 10-year occurrence of CVD showed higher specificities than sensitivities. However, these values are not directly comparable with our results due to the different selection of participants. Furthermore the differences may be explained by the different definition and cut-point for MA, different definition of outcome, and difference in length of follow-up-period and incidence of outcomes in the two populations.

The use of test performance measures in cross-sectional designs, for instance to diagnose an infectious condition, often yields higher values of specificity, sensitivity, PPV and NPV than we found in the present study. Of note, few tests use these measures to predict long term outcomes, and it is notoriously difficult to reliably predict future mortality and morbidity. MA is widely used in clinical work despite its seemingly modest predictive power, and 8-oxoGuo may well prove to be equally good and perhaps better than MA to predict long term outcomes if a more optimal cut-off value is identified. However, fine tuning of cut-off values was beyond the scope of this article. We used an arbitrary ≥ median 8-oxoGuo, and future research must further explore in this field.

An important limitation in this study is the reliance on a single measurement of the albumin and 8-oxoGuo in a morning spot urine sample. Glucose variability, which has been shown to exhibit a more specific triggering effect on oxidative stress than chronic sustained hyperglycemia [33], [34], was not assessed in the study. In addition, the potential impact of different treatment options on urinary albumin and 8-oxoGuo excretion was not determined. Finally, the lack of information on diet, for example on the consumption of antioxidants, which potentially could affect the level of oxidative stress, should be mentioned. The main strengths of our study include the large and population-based patient sample, the prospective design, the long follow-up period, and the low attrition rate.

As pointed out above, the exact mechanism underlying the association between albuminuria and CVD and death is unknown. This is also the case for 8-oxoGuo, but we have hypothesized that the oxidation of mRNA, rRNA, tRNA could affect the translational process negatively, leading to smaller quantities of protein overall and/or defective proteins, which may have detrimental effects on a variety of distinct cellular processes that lead to changes in proliferation and apoptosis and eventually the development of the atherosclerotic plaque [10]. Through this pathway increased RNA oxidation may be involved in the development of the vascular complications in diabetes.

In this study, both 8-oxoGuo and albuminuria were independent risk factors for mortality. Thus, both tests hold information about pathological processes beyond age, sex, comorbidity and the other covariates included in the multivariably adjusted model. We hypothesize that urinary albumin and 8-oxoGuo are proxy measures of different mechanisms involved in the development of complications, and by combining the two markers, we can get a more differentiated picture of the deteriorating processes that, if untreated, eventually will lead to overt CVD and death. The findings in this study suggest that measurement of urinary 8-oxoGuo provides additional information about risk to that obtained from urinary albumin, and that the combined use of the non-invasive biomarkers urinary 8-oxoGuo and urinary albumin could be useful for a better identification of patients at risk of CVD and death.

## Funding

This study was supported by the Research Committee at Copenhagen University Hospital – Rigshospitalet (Rigshospitalets Forskningsudvalg), the Research Committee at Bispebjerg Hospital (Bispebjerg Hospitals Forskningsudvalg), the Capital Region of Denmark (Region Hovedstaden), the Danish Medical Research Council, Aase and Ejnar Danielsen Foundation, P. Carl Petersen Foundation, the Augustinus Foundation, the Lundbeck Foundation, the Danish Research Foundation for General Practice, the Health Insurance Foundation, the Danish Ministry of Health, Novo Nordisk Farmaka Denmark Ltd., the A.P. Møller Foundation for the Advancement of Medical Science, and the Pharmacy Foundation.

## Duality of interest

The authors declare that there is no duality of interest associated with their involvement in this manuscript.

## Contribution statement

KB researched data, contributed to discussion, wrote the manuscript, and reviewed and edited the manuscript. NdFO was responsible for the original study design and did the data collection. FP, VS, RKR, NdFO and HEP researched data, contributed to discussion, and reviewed and edited the manuscript.
